# Microwave ablation of hepatic cyst: A case report

**DOI:** 10.1016/j.amsu.2020.11.058

**Published:** 2020-12-03

**Authors:** Radmila Vladimirovna Karpova, Daria Andreevna Petrenko, Anna Gevorikovna Saribekian, Andrey Andreevich Vedernikov, Muslim Magomedovich Magomedagaev, Andrey Sergeevich Gorbunov, Artem Anatolevich Shiryaev, Vladislav Konstatinovich Rybin, Kirill Fedorovich Chernousov

**Affiliations:** a Department of Faculty Surgery №1 of I.M. Sechenov, First Moscow State Medical University (Sechenov University), Russia; b International School «Medicine of Future» of Biomedical Park of I.M. Sechenov, First Moscow State Medical University (Sechenov University), Russia

**Keywords:** Microwave ablation (MWA), Hepatic cystadenoma, Malignancy risk, Telomerase

## Abstract

Hepatic cystadenoma is an urgent problem due to the high risk of malignant transformation. There are both radical and minimally invasive methods of treatment. We present a clinical case describing the successful use of microwave ablation (MWA) in a 72-year-old woman with hepatic cystadenoma. The patient was admitted to the clinic with abdominal discomfort, dull pain in the right hypochondrium, and weight loss of 10 kg during the previous year. The patient had a past medical history of liver cyst in segment VII. Ultrasound scanning, computed tomography (CT) of abdominal organs detected an increase in the size of the cyst, heterogeneity of its structure; the contrast enhancement was noted in the cyst wall. We suspected hepatic cystadenoma in segment VII and performed cyst puncture under ultrasound control – the obtained fluid revealed the presence of cylindrical epithelial cells, mucin, and macrophages in large quantities, high telomerase activity, CA 19-9 levels were greater than 1000 U/mL. Surgery was accomplished using the MWA catheter. Biological and cytological examination of the fluid confirmed the presence of signs of liver cystadenoma with a high malignancy risk. On the 2nd day after surgery ultrasound imaging of the abdomen revealed the residual cavity of 2 × 1 cm in segment VII. The patient was discharged with recommendations to conduct ultrasound examinations every six months. The control CT scan in 2020 showed no focal or cystic formations in the liver. In what way, MWA under control of ultrasound is a promising method of biliary cystadenoma treatment with high malignancy risk.

## Highlights

• Hepatic cystadenoma is an urgent problem due to the high risk of malignant transformation.
• There are few studies comparing the long-term results of microwave and radiofrequency ablation of hepatic cysts.
• Ultrasound-guided mini-invasive operations demonstrate high effectiveness for the treatment of malignant and benign tumors.
• MWA is considered as effective treatment for hemangiomas and non-parasitic hepatic cysts.
• Our clinical case shows great long-term results of using MWA in a 72-year-old woman with hepatic cystadenoma with high malignancy risk.
• MWA under control of ultrasound is a promising method of biliary cystadenoma treatment with high malignancy risk.

## 1 Introduction

This work has been reported in line with the SCARE criteria [1]. Biliary cystadenoma represents a benign, multilocular cystic neoplasm which is often lined by glandular mucin-producing epithelium. Biliary cystadenomas comprise 4–5% of all cystic lesions of the liver. Such cysts have a high malignant potential, and in some cases could cause cholestasis due to its size or position. Therefore, cystadenomas should be monitored in dynamics for timely surgical intervention if required. For many decades the segmental liver resection was considered as the treatment of choice for biliary cystadenomas but the appearance of the advanced methods, such as ablation (radiofrequency ablation (RFA), microwave ablation (MWA)), brought up doubts about previous treatment methods [2,3]. Researchers describing the advantages of minimally invasive techniques claim that in single-chamber small cysts in the liver, MWA is necessary, since in contrast to RFA, it creates a higher temperature and acts on a larger area, reducing the frequency of propagation of the process [4]. Numerous studies based on long-term results are required to compare the effectiveness of liver resection and ablation. Most scientists believe that surgical treatment cystadenoma liver depends on its size, multi-chamber, presence of telomerase activity (which indicates high risk of malignancy), diligence it to the major vascular structures of the liver and related diseases. The studies presented today demonstrate a small number of patients and the absence of long-term comparative results of microwave and radiofrequency ablation of the liver with high telomerase activity [5,6].

We present a ten-year follow-up of the patient after microwave ablation of biliary cystadenoma associated with the high risk of malignant transformation.

## 2 Сase presentation

**Fig. 1:**
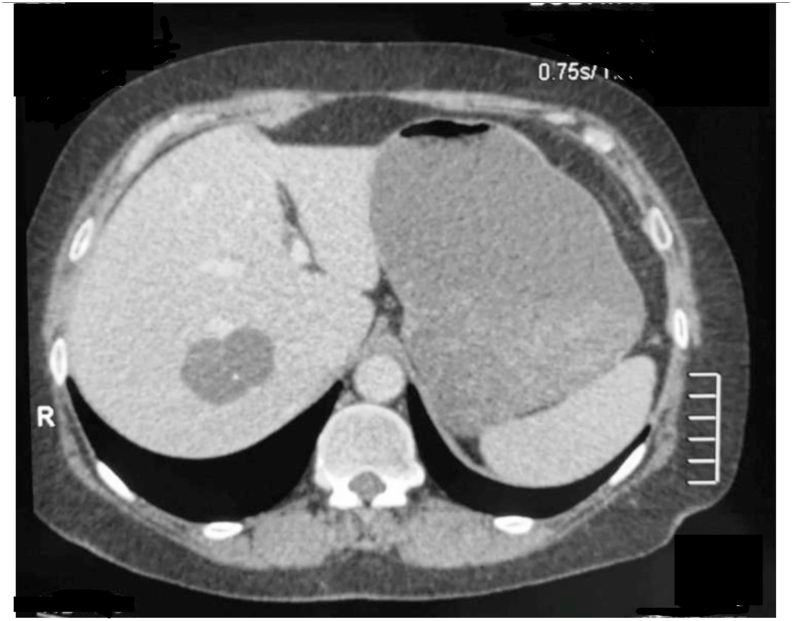
CT scan of the preoperative period. There is the dumbbell-shaped cyst of 4.3*3.1*3.7 cm with heterogeneous structure in the hepatic segment VII.

On March 16, 2011, the patient - a 72-year-old woman, was admitted to the Sechenov University Clinical Hospital No. 1 with abdominal discomfort, intermittent moderate dull pain in the right hypochondrium, and weight loss of 10 kg during the previous year. In 1991 the examination revealed a cyst with diameter of 1cm in the hepatic segment VII. It also showed cholelithiasis; the cholecystectomy was performed the same year. Subsequent dynamic monitoring of the cyst was not performed. In the fall of 2010 ultrasonography and computer tomography (CT) of abdominal organs detected the dumbbell-shaped cyst of 4.3*3.1*3.7 cm with heterogeneous structure in the hepatic segment VII; the contrast enhancement was noted in the cyst wall (Fig. 1).

On March 28, 2011, we performed cyst puncture under ultrasound control. Biological and cytological examination of the obtained fluid revealed the presence of cylindrical epithelial cells, mucin, and macrophages in large quantities. We also detected high telomerase activity, CA 19-9 levels greater than 1000 U/mL, and proteins level of 48 ppm. Rivalta's test was positive.

The diagnosis of biliary cystadenoma with high malignant potential was established. The patient was admitted to the Department of Surgery for the surgery.

Under intravenous anesthesia (Propofol 150 mg, Phentanylum 0.2 mg), an MWA catheter (MedWaves Incorporated, AveCure®) was inserted percutaneously into the cyst of the hepatic segment VII, accessing via the right anterior axillary line in the 10th intercostal space.

A percutaneous ultrasound-guided puncture of the biliary cyst of the hepatic segment VII was made using a 8Fr stylet catheter. After removal of the stylet, about 70 ml of a yellow transparent liquid was evacuated, which was sent for biological and cytological examination.

Then, a radiopaque preparation (Urografin®76% - 20 ml + NaCl 0.9% 50 ml) was injected into the cyst. During X-ray, a neoformation of 5 × 6 cm with clear, uneven contours was determined at the hepatic segment VII. The contrast spread into the abdominal cavity or the vessels was not detected.

**Fig. 2:**
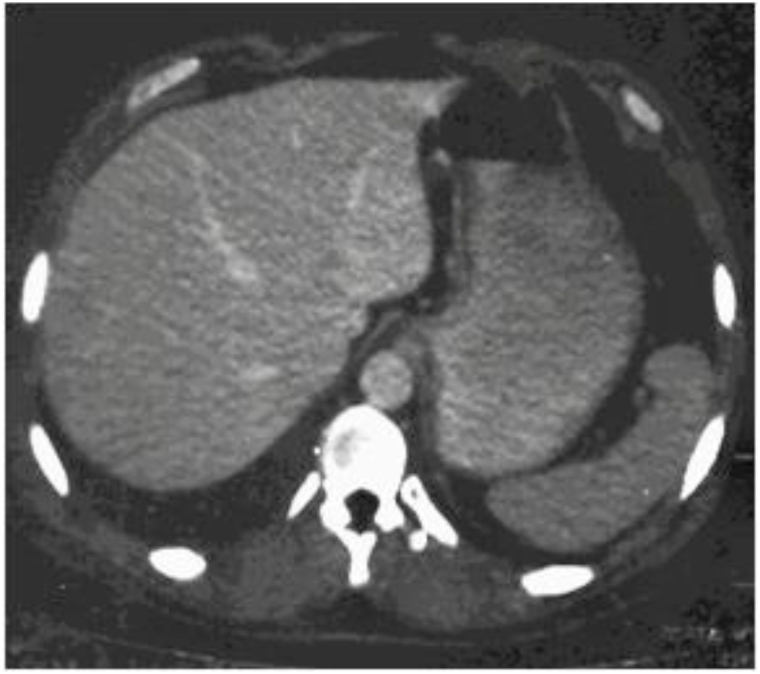
CT scan of the postoperative period. Absence of focal or cystic formations in the liver.

MWA of the cyst was performed using a MWA catheter placed in the cavity at the center of the cyst, with the frequency of 902–928 Hz delivered during 10 minutes. The inserted 8 Fr stylet catheter allowed the evacuation of the residual high temperature fluid during the MWA. Then the catheter and the needle were removed. Biological and cytological examination of the fluid obtained during the surgery confirmed the presence of biliary cystadenoma with a high malignancy risk. The postoperative period was uneventful, and no analgesics required. The abdominal ultrasound examination performed on the 2nd day after surgery revealed the residual cavity of 2 × 1 cm. The patient was discharged under the supervision of a general practitioner, with recommendations to refrain from physical activity for 3 months, and to conduct ultrasound examinations every six months. The patient underwent the control examination February 2020. CT scan of the abdominal cavity showed no focal or cystic formations in the liver (Fig. 2).

## 3 Discussion

Ultrasound-guided minimally invasive operations demonstrate high effectiveness for the treatment of malignant and benign tumors and gradually replace some open cavity operations. Such small interventions allow to achieve good treatment effects and positive long-term outcomes [7,8]. Recently, microwave ablation has drawn more attention as an effective treatment for malignant tumors smaller than 5 cm in diameter [9]. It was shown that MWA for hepatocellular carcinoma treatment demonstrated 43–60% 5-year survival rates and 24% 10-year survival rates [10]. MWA is considered as effective treatment for hemangiomas and non-parasitic hepatic cysts, since it allows to heat the cyst contents and its walls due to ultra-high-frequency (microwave) energy. This contributes to necrosis and sclerosis of the cyst walls. Moreover, by controlling the temperature and exposure time, using the generator it is possible to perform a complete destruction of the walls of the cyst [11]. Other advantages of MWA include the following: possibility to perform operation in open electrical circuit, without cooling (in contrast with RFA), and under local anesthesia; diminution in postoperative complications; and cost-efficiency due to the reduction in length of hospital stay.

According to the Liang P., Wang Y. clinical study of complications from percutaneous MWA, two deaths, found in a cohort of 1136 patients in 2009, were not directly related to MWA [12]. The main complications occurred in 30 (2.6%) patients and included liver abscess and empyema (n = 5), bile duct damage (n = 2), colon perforation (n = 2), tumor dissemination (n = 5), pleural effusion requiring thoracocentesis (n = 12), hemorrhage requiring arterial embolization (n = 1), skin burn requiring resection (n = 3). Some minor complications did not require treatment. As a result, it was concluded that MWA is a well-tolerated procedure with an acceptably low level of major complications in the treatment of malignant liver tumors [12].

Thus, taking into account positive, but few data on the effectiveness of MWA for HCC, we decided to use this method for the treatment of biliary cystadenoma with high malignant potential. The patient with cyst of 4.3*3.1*3.7 cm underwent MWA of the hepatic cyst under ultrasound control. There were no postoperative complications. The hospital stay was 3 days. We also obtained promising long-term results - CT scans, made 10 years later, showed no signs of cystic lesions in the liver.

## 4 Conclusion

MWA under control of ultrasound is a promising method of biliary cystadenoma treatment with high malignancy risk. Microwave ablation allows to achieve complete reduction of the epithelial lining of the cyst walls, its chambers, and also contributes to an uncomplicated postoperative period due to its minimally invasive and targeted technique of destruction of the pathological focus. The duration of the postoperative period is reduced and the positive long-term dynamics of patients increases.

## Ethical approval

The local ethics Committee of Sechenov University №10-19, Department of Faculty Surgery №1 of I.M. Sechenov, decided to approve the use of microwave ablation in a patient with liver cystadenoma. The authors are accountable for all aspects of the work in ensuring that questions related to the accuracy or integrity of any part of the work are appropriately investigated and resolved. the local ethics Committee №10-19 of Sechenov University, Department of Faculty Surgery № 1 of I.M. Sechenov.

## Source of funding

No sources of funding.

## Author contribution

All the authors made an equal contribution to the creation of the presented clinical case. Daria Petrenko, Anna Saribekian, Andrey Vedernikov and Muslim Magomedagaev, as 5th-year students, studied the patient's medical history in detail, analyzed the literature on nonparasitic liver cysts with a high risk of malignancy and surgical treatment options, compared the effectiveness of different methods of treating this pathology, and participated in writing the text. A team of surgeons from Sechenov University (Radmila Karpova, Andrey Gorbunov, Yuri Pavlov, Rybin Vladislav Konstantinovich and Kirill Chernousov) first applied the method of microwave ablation in the case of liver cystadenoma with high telomerase activity, guided the patient from the beginning of hospitalization and in the postoperative period, performed a control diagnostic study 10 years later, and participated in the writing of the article. Shiryaev Artem Anatolevich is surgeon and radiologist, the specialist of mini-invasive surgical methods in oncology gave us consultation and also participated in writing this article.

## Research Registration number

1. Name of the registry:

2. Unique Identifying number or registration ID:

3. Hyperlink to your specific registration (must be publicly accessible and will be checked):

## Guarantor

Karpova Radmila Vladimirovna.

## Consent

Written informed consent was obtained from the patient for publication of this case report and accompanying images.

## Provenance and peer review

Not commissioned, externally peer-reviewed.

## Declaration of competing interest

All authors have completed the ICMJE uniform disclosure form. The authors have no conflicts of interest to declare.
